# Characterising Biological and Physiological Drought Signals in Diverse Parents of a Wheat Mapping Population

**DOI:** 10.3390/ijms25126573

**Published:** 2024-06-14

**Authors:** Kamila Laskoś, Ilona Mieczysława Czyczyło-Mysza, Piotr Waligórski, Kinga Dziurka, Edyta Skrzypek, Marzena Warchoł, Katarzyna Juzoń-Sikora, Franciszek Janowiak, Michał Dziurka, Maciej T. Grzesiak, Stanisław Grzesiak, Steve Quarrie, Izabela Marcińska

**Affiliations:** 1 The Franciszek Górski Institute of Plant Physiology, Polish Academy of Sciences, Niezapominajek 21, 30-239 Kraków, Poland; k.laskos@ifr-pan.edu.pl (K.L.); p.waligorski@ifr-pan.edu.pl (P.W.); k.dziurka@ifr-pan.edu.pl (K.D.); e.skrzypek@ifr-pan.edu.pl (E.S.); m.warchol@ifr-pan.edu.pl (M.W.); k.juzon@ifr-pan.edu.pl (K.J.-S.); f.janowiak@ifr-pan.edu.pl (F.J.); michal.dziurka@gmail.com (M.D.); m.grzesiak@ifr-pan.edu.pl (M.T.G.); s.grzesiak@ifr-pan.edu.pl (S.G.); i.marcinska@ifr-pan.edu.pl (I.M.); 2 Faculty of Biology, University of Belgrade, Studentski trg 16, 11000 Belgrade, Serbia; steve.quarrie@gmail.com

**Keywords:** abscisic acid (ABA), biochemical parameters, drought, photosynthesis, wheat, yield

## Abstract

Water deficit affects the growth as well as physiological and biochemical processes in plants. The aim of this study was to determine differences in physiological and biochemical responses to drought stress in two wheat cultivars—Chinese Spring (CS) and SQ1 (which are parents of a mapping population of doubled haploid lines)—and to relate these responses to final yield and agronomic traits. Drought stress was induced by withholding water for 14 days, after which plants were re-watered and maintained until harvest. Instantaneous gas exchange parameters were evaluated on the 3rd, 5th, 10th, and 14th days of seedling growth under drought. After 14 days, water content and levels of chlorophyll *a*+*b*, carotenoids, malondialdehyde, soluble carbohydrates, phenolics, salicylic acid, abscisic acid (ABA), and polyamines were measured. At final maturity, yield components (grain number and weight), biomass, straw weight, and harvest index were evaluated. Physiological and biochemical parameters of CS responded more than those of SQ1 to the 14-day drought, reflected in a greater reduction in final biomass and yield in CS. Marked biochemical differences between responses of CS and SQ1 to the drought were found for soluble carbohydrates and polyamines. These would be good candidates for testing in the mapping population for the coincidence of the genetic control of these traits and final biomass and yield.

## 1. Introduction

Weather fluctuations play a pivotal role in governing crop growth and yields, particularly in rainfed agricultural systems [1,2]. The escalating issue of drought prompts widespread concern due to its adverse effects on both society and natural ecosystems, often categorized as a natural disaster [3,4]. According to Zhang et al.’s [5] meta-analysis on the impact of drought on rice and wheat agronomic traits, forthcoming droughts might lead to decreased yields compared to current ones. Moreover, the recent incidence of heatwaves, coupled with prolonged drought stress, poses potentially catastrophic consequences for cereal yields [6]. About 30% of all cereal cultivation area is taken up by wheat (*Triticum aestivum* L.), usually growing under abiotic stresses. An analysis of data from the years 1980–2015 reported up to 21% yield reductions in wheat on a global scale due to drought [7]. Plant sensitivity to drought depends on the intensity of the stress, the species, the genotype, and the developmental phase of the plant, as well as the presence of other stress factors [8]. Drought induces a number of morphological changes in plants, including the inhibition of stem elongation, root proliferation, leaf extension, and rolling [9,10]. Plants also show diverse physiological and biochemical reactions at cell and tissue levels aimed at coping with drought [9,11,12]. Physiological responses are associated with stomatal closure and a decrease in photosynthetic activity, which are manifested in changes in leaf gas exchange parameters [11]. Plants whose growth is limited by water shortage are characterized by low stomatal conductance without the modification of shoot water potential [13]. Severe drought also decreases chlorophyll content in leaves and causes damage in the photosynthetic apparatus [14]. These changes decrease the production and distribution of photoassimilates in the plant [13], inhibit leaf growth, and accelerate leaf senescence.

Phenolic compounds serve as powerful antioxidants because of their ability to eliminate free radicals [15,16]. Under drought, the efficiency of photosynthesis is positively correlated with a high concentration of phenolics (for example, ferulic acid) in the leaves [17,18]. Therefore, it is believed that a higher concentration of phenolic compounds in tissues can indirectly influence water relations, the functionality of the photosynthetic apparatus, the content of selected biochemical parameters, and plant yield productivity [19,20,21]. It was shown that high concentrations of cell wall-bound phenolics can be an important marker of drought susceptibility in plants, as water deficiency induces an increase in both phenolics content and antioxidant activity in cereals [22].

Abscisic acid (ABA) and salicylic acid (SA) are known as stress hormones which quickly accumulate under stress conditions and help plants survive the stress [10,23,24]. They enhance the activities of antioxidant defence systems [10,14,25], which lead to lower lipid peroxidation rates and consequently to the lower accumulation of malondialdehyde (MDA) [26], which is widely used as a marker for determining the degree of injury to a stressed plant [27]. Therefore, a lower MDA content indicates a higher antioxidative capacity, resulting in increased resistance to drought. Increased peroxidation can alter the physicochemical properties of membrane lipid bilayers, resulting in severe cellular dysfunction. The main role of ABA in the adaptation of plants to water deficit conditions is participation in stomatal closure, which reduces water loss through transpiration [28,29,30,31]. ABA is a master hormone in plants’ reactions to water deficit, and an elevated ABA level triggers the expression of numerous genes involved in these processes, which are controlled by ABRE (Abscisic Acid Response Element) sequences [32]. Another important stress hormone—SA—may decrease transpiration when applied exogenously [33] and inhibit ABA-induced stomatal closure. Sahu and Sabat [34] reported a negative effect of SA on plant growth and the activity of major enzymatic antioxidants. Okamoto et al. [35] discussed cross-talk between ABA and SA under stress conditions. The number of publications focusing on this aspect is increasing, but details of this cross-talk remain unclear.

Osmotic adjustment is one of the most significant mechanisms of drought tolerance in crops, helping plants with water uptake, which allows them to open stomata and assimilate CO_2_ even under soil drought conditions [12,23,36]. It has been observed that osmotic regulation is crucial in maintaining appropriate water content at low leaf water potential in many crops [12], including wheat [37,38]. Various nitrogen-containing compounds, including proline and polyamines (PAs), have been shown to accumulate under water restriction and facilitate the drought stress tolerance of wheat [39]. However, changes in PAs levels in reaction to drought stress are a much more complex phenomenon, with several authors describing a decrease in PAs levels on a different timescale under this stress [40,41]. PAs have been proposed as a new category of plant growth regulators involved in a large spectrum of physiological and biochemical processes and constituting an integral part of plant stress response [34,42,43,44]. The presence of several positively charged amino groups in PAs molecules gives them a polycationic character, enabling them to interact strongly with dipole molecules of water. PAs are able to bind with many biologically important molecules containing anionic groups, including nucleic acids, phospholipids, and proteins with various functions, such as membrane and receptor proteins, ion pumps, and enzymes, through strong ionic interactions with these structures, with PAs protecting them against the negative effects of dehydration on a microscale [45,46]. Moreover, PAs are a pool of substances involved in the mechanism of intracellular nitrogen-to-carbon balance and could function as a source of nitrogen for the synthesis of amino acids and also chlorophyll [47].

As different kinds of mechanisms are involved in plant responses to drought, it is important to establish a hierarchy of their impact on crop susceptibility to this stress to ensure yield maintenance, despite adverse environmental conditions [48,49]. In wheat breeding, enhancement for drought resistance might be accomplished using selection for favourable alleles of genes identified from quantitative trait loci (QTLs) for drought-related traits. Thus, identifying candidate genes at loci involved in drought resistance is an essential step in improving wheat drought resistance. QTL studies have been carried out in wheat using doubled haploid lines (DHLs) obtained by crossing the land race Chinese Spring (CS) in 1991 with the breeding line SQ1 [50,51,52,53]. Although QTLs for yield and its components have already been reported in these DHLs, grown in a range of environments including drought [50], the biological and physiological bases of drought responses determining variation in yield under drought have yet to be established. As Chinese Spring was subsequently selected as the first wheat genotype for genome sequencing [54], the serendipitous selection of CS as one of the parents of the mapping population makes these DHLs particularly useful for identifying candidate genes located within QTL regions regulating drought responses. Furthermore, the genome of SQ1 has recently been sequenced (Uauy, Simmons, and Adamski, unpublished), and gene polymorphisms between CS and SQ1 are available to facilitate the identification of candidate genes for biochemical and physiological traits associated with the better maintenance of yield and its components under drought.

Although CS and SQ1 were originally selected on the basis of the contrasting production of ABA in detached and partially dehydrated leaves (CS low and SQ1 high) [55], subsequent trials with CS and SQ1 plants grown in soil tubes and pots showed little difference in leaf ABA contents between CS and SQ1 in response to several days of stress (drought or salinity) [Quarrie and Steed unpublished, [56]]. Nevertheless, the parents are morphologically very diverse, with CS being tall and SQ1 having smaller leaves and spikes with awns (Figure S1) [57]. Apart from visible differences, CS and SQ1 are not yet well characterised at the biochemical and physiological levels, though Nergui et al. [58] recently concluded that CS has poor drought resistance characteristics. It is important to characterise upstream biochemical and physiological traits in the population’s parental genotypes as a starting point for further QTL studies to help characterise already-identified yield and component QTLs in terms of likely upstream biochemical and physiological drought responses which, in turn, determine how downstream yield components and hence yield itself are regulated under drought. Differences in biochemical and physiological characteristics between the two parental genotypes, mainly in their reaction to drought stress, provide an opportunity to evaluate dependencies between these key processes involved in the plants’ integrated reactions to drought stress.

Thus, the objective of the present study was to compare the responses of wheat genotypes CS and SQ1 to soil drought on the basis of key signalling molecules of drought perception to identify physiological and biochemical parameters to focus on in future QTL studies with the mapping population. In our previous studies, we characterized CS and SQ1 for their reactions to osmotic stress caused by polyethylene glycol (PEG) solution in hydroponic culture [59]. Here, we report the analysis of a diverse array of parameters (chlorophyll fluorescence, net photosynthesis, transpiration, stomata conductance, water use efficiency, total antioxidant activity, contents of stress signalling hormones abscisic acid and salicylic acid, total phenolics and sugar accumulation, membrane damage), as well as yield and its components (grain number, grain weight, biomass, shoot weight, harvest index), using plants grown in soil culture. These analyses allowed us to identify a network of dependencies between these parameters and major differences between CS and SQ1 in their drought responses.

## 2. Results

### 2.1. Physiological Changes

**Figure 1:**
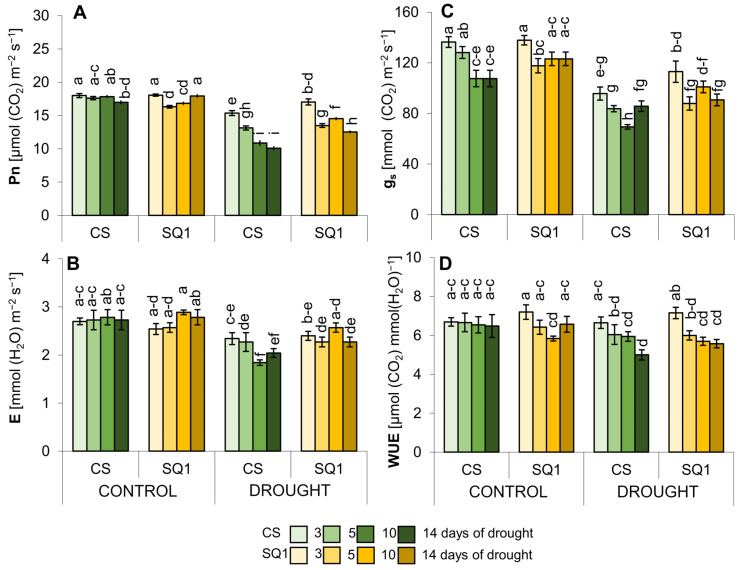
Gas exchange parameters: (**A**) net photosynthetic rate (Pn), (**B**) transpiration (E), (**C**) stomatal conductance (g_s_), and (**D**) water use efficiency (WUE) in Chinese Spring (CS) and SQ1 wheat cultivars depending on the treatment: control and drought. Mean values followed by the same letter are not significantly different (*p* ≤ 0.05). Error bars indicate SE (*n* = 7).

In terms of gas exchange and photosynthetic parameters, CS responded more to the drought treatment than SQ1, resulting in a greater reduction in net photosynthetic rate (Pn),transpiration (E), and stomatal conductance (g_s_) (Figure 1A–D). By the 3rd, 5th, 10th, and 14th day of withholding watering, Pn had decreased by 14, 25, 39, and 40% for CS and 5, 17, 13, and 30% for SQ1 in comparison with the control (Figure 1A). Thus, the net photosynthesis on day 14 of drought was significantly lower in CS than SQ1 (Figure 1A). Similarly, E had decreased significantly in CS by day 5 of the drought until its end, though E changed little in SQ1 during the drought treatment, with a significant reduction in SQ1 only on day 14 (Figure 1B). Conductance, g_s_, decreased significantly in both cultivars (Figure 1C), though the decreases were generally greater in CS (reductions of 30, 34, 34, and 18% for CS and 18, 23, 17, and 26% for SQ1). Drought-induced stomatal closure resulted in a moderate decrease in leaf water use efficiency (WUE) in both cultivars during the period of dehydration (Figure 1D), with a significant reduction observed only for CS on the 14th day of withholding watering: reductions of 18% for CS and 13% for SQ1.

The ANOVA of gas exchange showed significant differences between CS and SQ1 for most parameters but only under drought conditions (Table S1). The dominant source of variation was the number of days of withholding watering, except for E, which showed no significant effect of time for either cultivar. The interaction between cultivar and day showed that cultivars responded differently to measurement occasion for Pn under both treatments, E under drought, and g_s_ under drought. Despite the reduction in g_s_ under control conditions with time, especially evident in CS (Figure 1C), the ANOVA showed no significant cultivar x day interaction under control conditions.

### 2.2. Biochemical Changes

**Figure 2:**
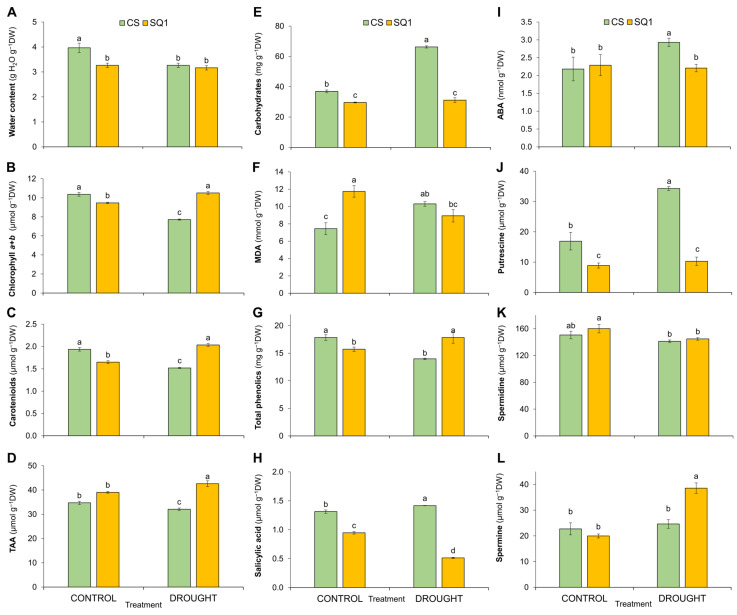
(**A**) Water content [g H_2_O g^−1^ DW]; content of photosynthetic pigments: (**B**) chlorophyll *a+b* [µmol g^−1^ DW] and (**C**) carotenoids [µmol g^−1^ DW]; (**D**) total antioxidant activity (TAA) [µmoles Trolox g^−1^ DW]; (**E**) soluble carbohydrates [mg g^−1^ DW]; (**F**) lipid peroxidation expressed as malondialdehyde (MDA) concentration [mmol g^−1^ DW]; (**G**) total phenolics [mg g^−1^ DW]; (**H**) salicylic acid [µmol g^−1^ DW]; (**I**) abscisic acid (ABA) [nmol g^−1^ DW]; and polyamines: (**J**) putrescine, (**K**) spermidine, and (**L**) spermine content [µmol g^−1^ DW] in Chinese Spring (CS) and SQ1 wheat cultivars depending on the treatment. Treatments were control and drought, measured on the 14th day of drought. Different letters indicate differences between genotypes according to the Duncan test (*p* ≤ 0.05). Mean values ± SE, *n* = 3. DW—the dry weight of leaves.

Biochemical changes in leaves are presented in Figure 2. After 14 days of withholding watering, leaf water content decreased more in CS than in SQ1 (Figure 2A). Total chlorophyll (Chl *a*+*b*) content in leaves decreased by 26% in CS and increased by 11% in SQ1 compared with corresponding controls (Figure 2B). Carotenoid (Car) contents also decreased by 21% in CS and increased by 23% in SQ1 (Figure 2C). After drought treatment, the total antioxidants activity (TAA), expressed in μmols of Trolox equivalents of small molecule antioxidants, decreased by 8% in CS while it increased by 9% in SQ1 compared with their controls (Figure 2D). Soluble carbohydrate contents increased by 79% in CS under drought stress, though these were unchanged in SQ1 (Figure 2E). Interestingly, under well-watered conditions, the content of MDA, which increases as damage to plasma cell membranes increases, was significantly higher (by 58%) in SQ1 than in CS (Figure 2F). Drought stress increased MDA content by 38% in CS and decreased it by 24% in SQ1. Quantities of phenolics under drought conditions decreased by 22% in CS, while they increased by over 13% in SQ1 (Figure 2G). In our study, SA content increased slightly (8%) but significantly in CS under water deficit conditions and decreased by 46% in SQ1 (Figure 2H). Compared with the corresponding controls, after water restriction, the content of ABA in CS increased by 34%, though it was unchanged in SQ1 despite 14 d of drought (Figure 2I). Three polyamines were measured in leaves: putrescine (Put), spermidine (Spd), and spermine (Spm) (Figure 2J–L). Although spermidine (Spd) contents were barely affected by drought in both CS and SQ1, for putrescine (Put) under drought conditions, its content doubled in CS, though it was unaffected in SQ1, while the opposite was true for spermine (Spm). In SQ1, under drought conditions, Spm increased by 93% compared with its control yet was unaffected by drought in CS.

**Figure 3:**
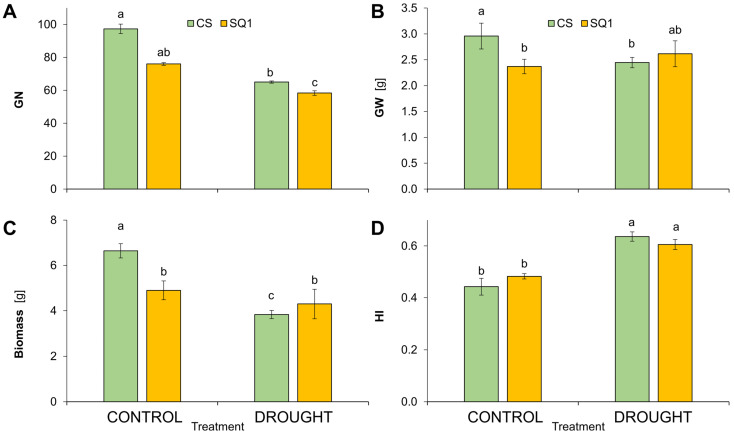
Grain number per plant (**A**), grain weight per plant (**B**), biomass (**C**), and harvest index (HI; (**D**)) in Chinese Spring (CS) and SQ1 wheat cultivars depending on the treatment: control and drought. Different letters indicate differences between cultivars according to the Duncan test (*p* ≤ 0.05). Mean values ± SE, *n* = 3.

Following the drought treatment, significant differences were observed in the number of grains (GN) and their weight (GW) per plant (Figure 3). Drought decreased GN by 34% in CS and 23% in SQ1 (Figure 3A) and decreased GW significantly by 13% in CS. However, GW was not significantly affected by drought in SQ1 in comparison with its controls (Figure 3B). After withholding watering, biomass decreased by 42% in CS but only 12% in SQ1 in comparison with their controls (Figure 3C). Harvest index, calculated as the ratio of seed weight to total above-ground weight per plant, increased significantly under drought stress—by 45% in CS and 27% in SQ1 in comparison with the control (Figure 3D). Thus, the drought treatment had no effect on the yield (GW) of SQ1, though GW was significantly decreased by drought in CS. Biomass and straw per plant were highly correlated (0.93 correlation coefficient), so data for straw weight are not included in Figure 3.

As with gas exchange parameters, the ANOVA for biochemical traits, yield, and agronomic components also showed a significant influence of the cultivar (for 13 of 17 traits) and the drought treatment (for 12 of 17 traits) (Table S2). The large number of highly significant interaction terms (for 14 of 17 traits) demonstrated that CS and SQ1 responded differently to the drought treatment not only for gas exchange parameters but also for many biochemical, yield, and agronomic traits.

**Figure 4:**
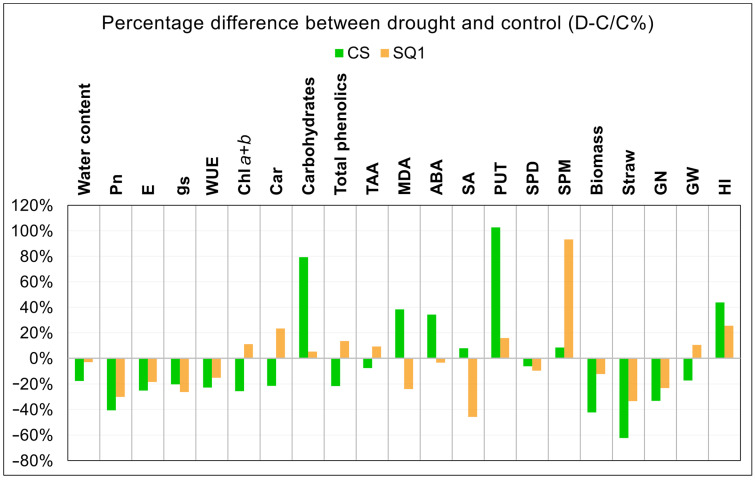
Percentage differences between 14-day drought stress (D) and control (C) for all parameters measured in CS and SQ1 plants.

To clarify the picture of differences between CS and SQ1 in responses to the 14-day drought treatment, Figure 4 shows percentage differences between control and 14-day drought treatments for the two cultivars and all parameters measured, ranked from instantaneous measurements of gas exchange parameters to integrated yield and agronomic traits. Here, similarities between CS and SQ1 in the responses of gas exchange parameters to drought are evident, and these are largely reflected in long-term effects on plant biomass, straw, and grain number. Apart from antioxidants (Trolox) and spermidine (Spd), all other biochemical traits showed differences between CS and SQ1 in drought responses of at least 35%, with drought stimulating either large increases or large decreases in biochemical trait concentrations depending on the cultivar and trait. Overall, drought had a greater effect in reducing agronomic and yield traits of CS than those of SQ1.

## 3. Discussion

Many physiological processes are affected by water shortage. As water provides the environment for all chemical and physical processes taking place in the plant, its deficiency leads to a serious disruption in their progress. Considerable changes occur in a wide range of processes including photosynthesis, transpiration, antioxidant activity, the accumulation of osmoprotectants, and yield productivity. In our study, we used wheat cultivars initially selected to differ specifically in their profiles of ABA accumulation in response to drought stress in detached leaves (CS-low ABA, SQ1-high ABA). Previous work in field trials using plot shelters with wheat lines segregating from a cross to differ in ABA accumulation using this detached-leaf test had shown significant differences in response to both a 4-week pre-anthesis drought and a terminal drought from anthesis to maturity [60]. High-ABA lines significantly outyielded low-ABA lines and had greater crop water use efficiency. SQ1 was subsequently selected from the highest of these high-ABA lines tested in the drought trials. Although further analyses of leaf ABA contents in stressed plants showed no significant differences between ABA contents of CS and SQ1, these lines were known to differ in major morphological traits (Figure S1). CS was also shown to have more surface roots but fewer roots at depth than SQ1 [Quarrie and Steed, unpublished RASP report]. As a mapping population of DHLs is available from the cross CS × SQ1 [50], studying the parents can give valuable information on traits associated with drought stress that have intermediate roles in determining the yield responses of wheat to drought stress.

After subjecting the plants to a short-term drought stress during the vegetative phase of development, we measured parameters relating to several physiological and biochemical processes to establish likely interrelations between them and subsequent long-term consequences for yield and agronomic traits.

Photosynthesis is the most important process taking place in the plant, thanks to which the plant obtains the necessary energy and carbon. Each disturbance of photosynthesis has a considerable impact on the course of all physiological processes [61]. Our results demonstrated that drought significantly reduced net photosynthesis, transpiration, and stomatal conductance in both cultivars, though leaf water content decreased more in CS than in SQ1 (Figure 4). There was a clear trend in both genotypes for WUE to decrease, and this reached significance in CS on day 14. Similar results were obtained by Xu and Zhou [62]. This is also in agreement with our previous investigation comparing CS and SQ1 using hydroponic culture [59], where it was found that a greater decrease in net photosynthetic rate in CS compared with SQ1 under drought was associated with changes in biochemical processes affected by lipid peroxidation.

Prasad et al. [63] suggested that high chlorophyll content is a very important trait for grain production. Our spectrophotometrical measurements showed that chlorophyll *a*+*b* as well as carotenoid content under drought treatment decreased significantly in CS but increased slightly, but significantly, in SQ1 compared with their controls. This phenomenon can be explained by the hypothesis that a greater leaf water loss after 14 days of drought in CS plants led to oxidative stress by inhibiting gas exchange through stomatal closure, resulting in excess energy in the chloroplast, which could not be used for the Calvin cycle [64]. Consequently, reactive oxygen species (ROS) accumulated. Of course, this accumulation would trigger ROS-scavenging systems, but, if drought stress is prolonged, ROS production would overwhelm the scavenging action of the antioxidant system, resulting in extensive cellular damage. An excess of ROS destroys chlorophylls and carotenoids, and concentrations of these significantly decreased in CS after 14 days of drought (Figure 2). This hypothesis is supported by the significant decrease in total antioxidant activity in CS under drought, while in SQ1, antioxidant activity slightly increased. 

Drought had a very similar effect on total antioxidant activity and phenolics levels, which is understandable as these compounds account for a significant part of the antioxidant capacity, which is therefore strongly correlated with total phenolic content [65]. An additional aspect of the accumulation of phenolics under drought stress is that these compounds can act as low-molecular-weight osmoprotectants and thus, together with proline and carbohydrates, they are expected to help in the regulation of osmotic fluctuation under drought. This is an additional side effect of ROS accumulation under drought stress. Moreover, Hura et al. [20] demonstrated that the incorporation of phenolics in the cell wall is mediated by hydrogen peroxide. Hence, an increase in the content of cell wall-bound phenolics is accompanied by the more effective scavenging of H_2_O_2_. Another result confirming the above-described mechanism of oxidative damage was the accumulation of MDA (used as a biomarker of the degree of oxidative stress) in drought-treated CS plants, while the opposite reaction was observed in SQ1 plants. This phenomenon in CS indicates the intensified oxidation of biological membranes caused by ROS [66]. An increase in MDA has been presented before in studies where plants were exposed to abiotic stress, for example, Ma et al. [67] in wheat. Nevertheless, SQ1 had 58% more MDA than CS in the control treatment, which is difficult to reconcile with both CS and SQ1 being well-watered through the experiment. However, Khoubnasabjafari et al. [68] questioned the use of MDA as a biomarker of oxidative stress, pointing out that results can vary significantly according to the effects of procedural modifications on MDA-TBA adduct development and the low stability of MDA in biological samples. Therefore, our findings on MDA variation amongst both cultivar and treatment should be regarded with caution.

According to Aslam et al. [31], drought at the early stage of plant development could trigger early flowering and reduce tiller numbers through the accumulation of ABA by activating ABA signalling components that control water status and stomatal closure, promoting plant escape or adaptation to drought stress. We recorded substantial decreases in transpiration and stomatal conductance during the drought treatment, which was most likely an effect of elevated ABA level under these conditions, as this has been observed previously in wheat [69,70,71]. This decrease in stomatal conductance was particularly marked in CS, which showed significantly increased leaf ABA content after 14 d of drought. Although SQ1 showed no increase in whole leaf ABA content after 14 d of drought, the reduction in transpiration and conductance in SQ1 could have resulted from the redistribution of ABA to the epidermis or increases in ABA content present in earlier phases of the drought treatment.

A mechanism to maintain leaf functioning during drought stress is osmotic adjustment (the accumulation of low molecular weight osmolytes to maintain leaf turgor) [72]. Munns et al. [73] also found that osmolytes influence changes in osmotic potential in the apex and young leaves of wheat seedlings growing in conditions of water deficit, mainly due to the accumulation of soluble carbohydrates and amino acids. Our study showed a much higher concentration of carbohydrates in CS compared with both the control treatment and SQ1, and this may have supported a higher osmotic adjustment in CS than in SQ1. However, this accumulation of carbohydrates did not prevent Pn from decreasing during the drought. Indeed, the high leaf soluble carbohydrate contents were probably associated with the reduced export of carbohydrates to the developing leaves and ear, leading to smaller plants and fewer floral organs at the anthesis and therefore lower grain numbers in CS than in SQ1. The drought treatment caused a much bigger decrease in plant biomass in CS than in SQ1 (Figure 4), which would have been a consequence of the restricted export of carbohydrates from the leaves of CS.

Osmotically active compounds also include proline and polyamines (PAs), but the observed concentrations of these compounds were undoubtedly too low compared with the main osmoprotectants, such as soluble sugars and mineral salts (osmotic pressure being a colligative property of solutions, i.e., it does not depend on the chemical property of an osmotically active compound but on its concentration). It is known that some osmoprotective compounds accumulate in specific plant organs, compartments or cellular structures, acting locally and not on the scale of the whole organism [74]. Polyamines can associate with cell membrane components, e.g., phospholipids, to protect lipid bilayers from the destructive influence of drought [75]. Some studies have shown that levels of the three most frequently occurring polyamines—Put, Spd, and Spm—significantly increase under abiotic stress [76]. In our study, CS and SQ1 differed markedly in the responses of these three polyamines to drought stress. The content of Put increased under drought in CS and Spm increased in SQ1, while the level of Spd decreased slightly in both CS and SQ1, though the decrease was significant only for SQ1. Although Islam et al. [77] found a difference in the responses to salt stress between putrescine and spermine in rice, Hassan et al. [78] found both PAs to be equally effective in protecting wheat plants from drought stress. Therefore, the physiological consequences of these large differences between CS and SQ1 in the PAs they accumulated is yet to be determined. Although the signalling function of polyamines and free proline in plants subjected to drought stress is not well-documented, the accumulation of free proline is usually a good marker of stress development in plants [79]. Although free proline contents in CS and SQ1 were also measured in this experiment, results are not reported here, as free proline has been measured on several other occasions in CS and SQ1 under well-watered and droughted conditions, and results have varied considerably from experiment to experiment. For example, under well-watered (control) conditions, leaf proline contents varied amongst five experiments from 0.11 to 0.97 mg/g DW in CS and from 0.12 to 0.80 mg/g DW for SQ1. Although in general, drought stimulated an increase in free proline concentrations (0.18–6.58 mg/g DW), replicate plant-to-plant variation was considerable, and proline concentration was not obviously associated with the level of drought stress applied. Some of this variation may have been due to considerable diurnal variation in free proline, as reported by Treichel et al. [80] and Batanouny et al. [81]. Thus, we do not believe that free proline concentrations are a reliable predictor of other physiological and biochemical responses of, at least, these wheat cultivars to drought stress.

The significant decrease in water content observed under drought stress is the basic physicochemical factor initiating the above-mentioned secondary reactions, with the entire sequence of events triggered and controlled by signalling factors. Theoretically, this role can be played by any factor whose intensity varies under the influence of a trigger and which can operate remotely. The signalling factors discussed most often are chemical in nature, such as free proline, polyamines, SA, and ABA. They are water-soluble, low-molecular-weight compounds, the concentrations of which change significantly under drought stress but remain relatively low. Although SA influences a variety of signalling pathways in plant defence against abiotic stresses and modulates plant response to drought to induce diverse stress tolerance mechanisms, these effects have been established almost exclusively by the exogenous application of SA. Very few studies are available showing the effect of drought stress on leaf SA contents. Thus, Abreu and Munné-Bosch [82] showed an approximately 30%, but non-significant, increase in leaf SA contents in sage after 14 days of drought stress, and Malaga et al. [83] showed SA contents in leaves of doubled haploid barley lines to either increase or decrease in response to drought stress, depending on the line. In our study, SA content in CS increased slightly but significantly in water deficit conditions, whereas it decreased markedly in SQ1 under the same circumstances. In view of the scarcity of reports on endogenous levels of SA in plant leaves during drought stress, the physiological consequences of these SA differences between drought responses of CS and SQ1 are unclear.

Generally, ABA is regarded as the main physiological trigger for stomata closure, which prevents water loss by reducing transpiration [84], as well as a cascade of other physiological and biochemical changes (e.g., [32]). The obtained results show that net photosynthesis in SQ1 under drought conditions was generally higher than in CS, and as the drought severity developed, the decrease in net photosynthesis in SQ1 was significantly less than in CS. The greater reduction in CS (in comparison with SQ1) may have been due to a larger increase in leaf ABA contents in CS than in SQ1 as drought developed, though leaf ABA contents were measured only after 14 days of drought, by which time ABA content was still significantly increased in CS, while in SQ1, ABA contents were similar in both control and droughted plants. This could be explained by the lower degree of stress after 14 days in SQ1, as recorded by leaf water content (Figure 4).

Drought stress is the main environmental stress limiting the development and yielding of cereals [85] through disturbances in ear development, the pollination process, and then seed setting [86]. Water deficit also causes phenological changes associated with a reduced number of tillers and spikes, fewer spikelets per spike, and reduced spikelet fertility, which consequently lead to seed reduction [87]. In our studies, although CS was better-yielding that SQ1 under irrigated conditions (Figure 3), yield (grain weight) was significantly reduced by drought in CS, whereas SQ1 was able to maintain its yield under the drought treatment, despite a significant reduction in grain number in both SQ1 and CS. These differences between CS and SQ1 were reflected in decreases in biomass and straw weight under drought stress, which were large in CS but much smaller in SQ1. The larger reductions in biomass than yield resulted in higher HI in both cultivars. In a study by Hou et al. [88], grain yield was higher in a wheat cultivar with better physiological responses to drought when compared with another cultivar, which shows that a plant that is more resistant to drought with better photosynthetic capacity may mitigate the decrease in yield under these stress conditions. Prasad et al. [63] observed that drought negatively affects the setting of seeds and their weight, and drought-induced increases in HI through increased carbon distribution to seeds were reported by Monneveux et al. [89], despite a reduction in shoot weight.

## 4. Materials and Methods

### 4.1. Plant Material

Seeds of two cultivars of hexaploid wheat were obtained from the John Innes Centre, Norwich, UK. Cultivars Chinese Spring (CS) and SQ1—a line bred for high ABA levels in detached and partially dehydrated leaves, according to Quarrie et al. [50]—were used in the experiment. The breeding line SQ1 was selected at the John Innes Centre, Norwich, UK, from the 7th cross generation (F7) between two wheat cultivars: Highbury x TW269/9/3/4.

### 4.2. Growth Conditions

**Figure 5:**
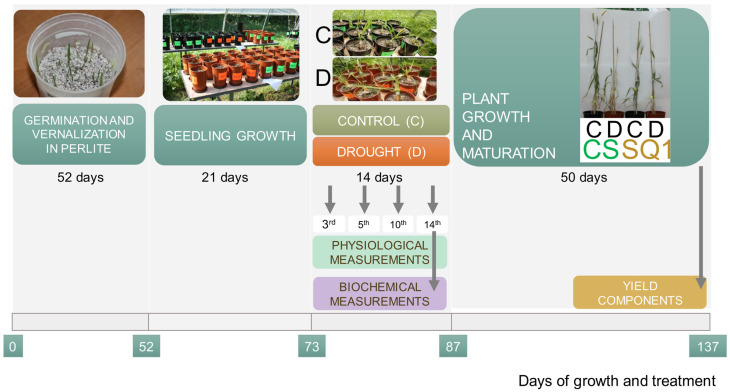
Scheme of the experimental set-up for CS and SQ1 wheat cultivars, with drought stress induced by withholding water supply for 14 days during growth in the soil. C—control, D—drought.

Seeds of CS and SQ1 were germinated and vernalized for 7 weeks at 4 ± 1 °C with an 8/16 h light/dark photoperiod at a light intensity of 150 μmol m^−2^ s^−1^. Twenty seedlings of each genotype were then individually planted into 3 dm^3^ pots with a mixture of horticultural soil and sand (1/1 *v*/*v*). Before and after drought application, every week, plants were watered with Hoagland and Arnon medium [90]. Soil water content was measured with a Hydro Sense Soil Water System CS630 (Campbell Scientific, Logan, UT, USA) using 12 cm probes and maintained at 23 ± 3% of soil volumetric water content (VWC). Plants were transferred to an open-sided vegetation tunnel and grown until maturity. Drought stress, induced by withholding water supply, was applied to one half of the CS and SQ1 plants with five expanding leaves on the main shoot (rapid tillering phase). Irrigation was withheld for 14 days until the leaves showed visible signs of turgor loss and VWC reached 8 ± 1%. For the control, VWC was kept at 23 ± 3%. Then, all plants were re-watered to 23 ± 3% and maintained until harvest. The scheme of the experiment is presented in Figure 5.

### 4.3. Measurements and Analysis

Diverse physiological and biochemical parameters were measured. Gas exchange parameters (Pn, E, g_s_, WUE) were measured on the 3rd, 5th, 10th, and 14th day of withholding water supply. Non-destructive measurements (photosynthetic activity) were conducted on seven replicate plants per cultivar, treatment, and occasion. Samples were collected only at 14 d after withholding water to measure leaf water content, antioxidant activity (in Trolox equivalents), and lipid peroxidation (MDA), as well as the content of soluble carbohydrates, phenolics, polyamines (putrescine, spermidine, spermine), salicylic acid, abscisic acid, and photosynthetic pigments (chlorophyll *a*+*b,* carotenoids). Measurements of biochemical parameters were performed on the fourth and fifth fully expanded leaves combined for each plant. Combined leaves were frozen in liquid nitrogen and stored at −70 °C then lyophilized and homogenized to a powder to determine biochemical parameters. Samples were taken for ABA from three replicate plants per treatment and cultivar and from another three replicate plants per treatment and cultivar for other biochemical parameters. 

Water content in leaves on day 14 was determined as H_2_O content by weighing leaves 4 and 5 combined from three plants per treatment and cultivar before and after lyophilisation using the following formula: (FW − DW)/DW, where FW is the fresh weight and DW is the dry weight of leaves.

Remaining plants (with leaves 4 and 5 intact) were sampled at maturity for yield and agronomic parameters with three replicate plants per cultivar and treatment.

### 4.4. Physiological Parameters

Gas exchange measurements were performed using an infrared CO_2_ analyser IRGA (CI-301PS, CID, Camas, WA, USA). Net photosynthetic rate (Pn) [µmol (CO_2_) m^−2^ s^−1^], transpiration rate (E) [mmol (H_2_O) m^−2^ s^−1^], and stomatal conductance (g_s_) [mmol (CO_2_) m^−2^ s^−1^] were determined under air flow of 200 mL min^−1^, saturating PPFD of 1100 μmol (photons) m^−2^ s^−1^ provided by an LED light module (CI-301LA, CID, Camas, WA, USA), and air CO_2_ concentration of 380 ppm provided by a CO_2_ control unit (CI-301AD, CID, Camas, WA, USA). Coefficient of water use efficiency (WUE) was calculated based on the measurements of Pn and E (Pn/E).

Chlorophyll (Chl) and carotenoids (Car) contents were determined in 5 mg of powdered leaves extracted in 1 mL of 80% ethanol and centrifuged at 2000× *g* (Universal 32R, Hettich, Germany) for 10 min. The absorbance of 200 µL supernatant was measured at 470, 648, and 664 nm on a microplate reader (Synergy 2, Bio-Tek, Winooski, VT, USA). The concentrations of chlorophyll *a*, *b* and carotenoids were determined according to the following equations:

Chl *a* (mg/mL) = 12.7 × A664 − 2.7 × A648

Chl *b* (mg/mL) = 22.9 × A648 − 4.7 × A664

Car (mg/mL) = (1000 × A470 − 2.13 × Chl a − 97.64 × Chl b)/209

where Chl *a* = chlorophyll *a*, Chl *b* = chlorophyll *b*, Car = carotenoids, A470 = absorbance at 470 nm, A648 = absorbance at 648 nm, and A664 = absorbance at 664 nm.

### 4.5. Biochemical Parameters

The procedures for measurement of biochemical parameters (content of soluble carbohydrates, total phenolics, salicylic acid, abscisic acid, and endogenous polyamines) were described in our previous papers [59,91]. The content of soluble carbohydrates was measured spectrophotometrically. Leaves were extracted in ethanol and the procedure was carried out according to Dubois et al. [92]. Absorbance of the solution was measured at 490 nm with a microplate reader (Synergy 2, BioTek, Winooski, VT, USA). For evaluation of total phenolics contents, leaves homogenized in ethanol were prepared according to Singleton and Rossi [93]. Solutions supplemented with Folin–Ciocalteu reagent were measured at a wavelength of 760 nm. Salicylic acid (SA) was extracted with cold methanol according to Wilbert et al. [94], modified by Dziurka et al. [95]. SA content was measured using the HPLC system Agilent 1260 (Agilent, Waldbronn, Germany) equipped with a 6420 ESI tandem mass spectrometer (Agilent, Santa Clara, CA, USA). The column used was Supelco Ascentis RP-Amide 75 × 4.6 mm 2.7 μm (Supelco Inc., Bellefonte, PA, USA), with gradient elution using water with 0.1% formic acid (A) and water/methanol (1:1 *v*/*v*) (B). The content of abscisic acid (ABA) was determined using indirect enzyme-linked immunosorbent assay (ELISA) according to Walker-Simmons and Abrams [96]. The ABA antibodies were derived from MAC 252 (Babraham Technix, Cambridge, UK). Absorbances were measured at 405 nm with a spectrophotometer (Model 680, Bio-Rad Laboratories, Hercules, CA, USA). The activity of low-molecular-weight antioxidants in the tissues was measured using the DPPH method according to Brand-Williams et al. [97] with modifications by Laskoś et al. [98]. The results were expressed as μmols of Trolox equivalents. The contents of polyamines were determined by HPLC after dansylation [99]. Lipid peroxidation was also determined as the concentration of malondialdehyde (MDA) according to Dhindsa et al. [100]. The specific absorbance of the extract was measured at 532 nm.

### 4.6. Yield Components and Biomass

At full plant maturity, grain number per plant, grain weight per plant, biomass (shoots with spikes), and straw (biomass minus grain weight per plant) were determined for each plant. Harvest index (HI) was also calculated as the quotient of grain weight to biomass weight. 

### 4.7. Statistical Analysis

The results presented in figures and tables constitute mean values ± standard error (SE) based on seven plants as replicates for non-destructive measurements and on three replicate plants for biochemical and yield measurements. Data were analysed using an ANOVA and Duncan’s multiple range test at *p* ≤ 0.05 with the statistical package STATISTICA 13.0 (TIBCO Software Inc. Palo Alto, CA, USA).

## 5. Conclusions

The objectives of this study were to characterise the parents of a mapping population of wheat doubled haploid lines from the cross Chinese Spring x SQ1 grown in soil and given a short-term drought treatment to stimulate physiological and biochemical responses. Although the genetic control of yield and its components in the mapping population has already been reported [50], the work described here will allow differences in yield amongst the mapping population to be dissected through intermediate physiological and biochemical processes to help determine the primary effect of candidate genes for the differences between CS and SQ1 allele effects on yield.

Thus, we have confirmed that the taller CS plants have greater biomass than SQ1 plants under control conditions, though the 14-day drought during the vegetative phase had a greater effect on reducing the final biomass of CS than SQ1. This was reflected in a greater yield reduction following the drought stress in CS than in SQ1, especially regarding grain number per plant. Evidently, CS was not able to recover from the short-term drought by compensatory biomass accumulation post-drought. In contrast, SQ1 was able to recover its growth after rewatering plants, so that both final biomass and yield were not significantly different between treatments.

Both cultivars responded to the 14-day drought by reducing gas exchange parameters, as well as chlorophyll and carotenoids, though increasing drought stress reduced Pn more in CS than in SQ1, which was associated with the considerable retention of soluble carbohydrates in CS. This growth reduction during drought may have prevented CS from recovering its growth following stress relief. The greater instantaneous and long-term biomass reduction in CS than in SQ1 was reflected in the differing responses of the cultivars to measures of oxidative stress (MDA and total antioxidant activity) and especially the major stress hormone ABA. CS and SQ1 also differed markedly in the responses of PAs to drought stress. 

For future genetic analysis using the CSxSQ1 mapping population, our findings reported here on large drought-induced differences between CS and SQ1 in, particularly, leaf soluble carbohydrates, putrescine and spermine, make these good candidates for study in the mapping population to test coincidences of quantitative trait loci (QTLs) for these biochemical traits with QTLs for final biomass and yield components. The genomes of both CS and SQ1 have now been sequenced and gene polymorphisms are available, which will facilitate the identification of candidate genes for coincident trait QTLs.

## Data Availability

All data are contained within the article. The datasets used and analysed during the current study are available from the corresponding author on reasonable request.

## Supplementary Materials

The following supporting information can be downloaded at: https://www.mdpi.com/article/10.3390/ijms25126573/s1.
