# Supplementary material for: Characterising Biological and Physiological Drought Signals in Diverse Parents of a Wheat Mapping Population
Source: Int J Mol Sci. 2024 Jun 14;25(12):6573. doi: 10.3390/ijms25126573 (PMC11203422; doi:10.3390/ijms25126573)
Supplement: Supplementary file 1 [file ijms-25-06573-s001.zip › Figure S1.pdf]

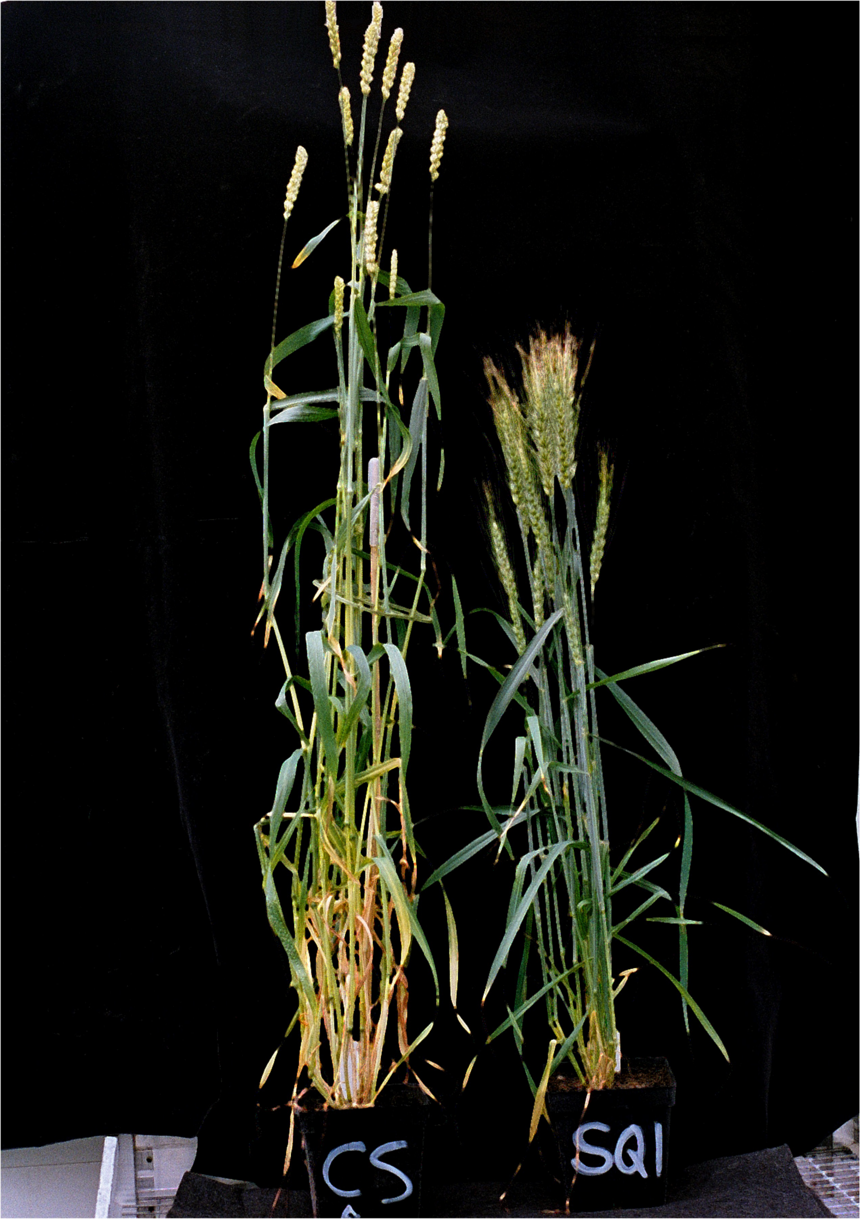

Figure S1. Representative plants of Chinese Spring and SQ1 during grain filling, showing contrasting morphology of the two cultivars.
