# Supplementary material for: Characterising Biological and Physiological Drought Signals in Diverse Parents of a Wheat Mapping Population
Source: Int J Mol Sci. 2024 Jun 14;25(12):6573. doi: 10.3390/ijms25126573 (PMC11203422; doi:10.3390/ijms25126573)
Supplement: Supplementary file 1 [file ijms-25-06573-s001.zip › Table S1.pdf]

Table S1. F-ratios and significance levels of gas exchange parameters: net photosynthetic rate (Pn), transpiration (E), stomatal conductance (g_s) and water use efficiency (WUE) in CS and SQ1 wheat cultivars under control (C) and drought (D) conditions. *,**,*** indicate significance at $p \leq 0.05$, 0.01, 0.001, respectively.

**Trait**	**Treatment**	**Between cultivars**	**Between days**	**Interaction cult x day**
Pn	C	2.1	4.0*	5.4**
	D	117.7***	120.3***	13.6***
E	C	0.2	1.0	0.5
	D	10.0**	1.3	4.3**
g_s	C	2.1	7.4***	2.8
	D	15.9***	6.4**	3.0*
WUE	C	0.1	1.2	0.9
	D	0.9	10.5***	0.9
