# Supplementary material for: Characterising Biological and Physiological Drought Signals in Diverse Parents of a Wheat Mapping Population
Source: Int J Mol Sci. 2024 Jun 14;25(12):6573. doi: 10.3390/ijms25126573 (PMC11203422; doi:10.3390/ijms25126573)
Supplement: Supplementary file 1 [file ijms-25-06573-s001.zip › Table S2.pdf]

Table S2. F-ratios and significance levels of biochemical traits and selected yield components for CS and SQ1 wheat cultivars measured in control (C) and drought treatment (D). *, **, *** indicate significance at $p \leq 0.05, 0.01, 0.001$, respectively.

**Trait**	**Between cultivars**	**Between treatments**	**Interaction**
Chlorophyll *a*+*b*	53.08***	38.64***	202.69***
Carotenoids	14.04**	0.26	173.99***
Malondialdehyde	7.27*	1.53	16.98**
Soluble carbohydrates	471.01***	249.93***	202.22***
Total antioxidants	111.62***	0.53	24.80***
Proline	1578.66***	81.87***	349.23***
Total phenolics	1.98	2.01	23.45**
Salicylic acid	1170.65***	79.04***	209.04***
Abscisic acid	7.97*	0.03	11.42**
Putrescine	90.12***	30.98***	22.38**
Spermidine	2.20	7.71*	0.43
Spermine	9.49*	32.09***	21.21**
Grain number	15.88*	32.00**	0.40
Grain weight	0.98*	0.75*	0.01
Biomass	2.26	16.02**	6.76*
Straw weight	3.24*	18.43***	8.54**
Harvest index	1.16	95.78***	7.86*
